# Why Not

**Published:** 1882-08

**Authors:** 


### WHY NOT.

At the approaching meeting of the American Dental Association there should be some conference by the faculties or representatives of the various dental colleges of this country. Some twelve years ago there was formed an association of the faculties of the dental colleges in the United States.

For a time the results of its work were good, in promoting a better feeling and in elevating the standard of dental education. After several successful meetings some unfortunate circumstances took place, that impaired the usefulness of the association, so that its meetings ceased to be held. But with increased number of colleges and the awakened interest in the subject of dental education; it would seem eminently fit that such an association should be organized and made to subserve the interests of the profession, as well as similar organizations promote the welfare of general education, and some of the special systems of education. The medical faculties of this country have their association, through which much is accomplished.

Such an association of the faculties of the dental colleges would bring the members to a better acquaintance with each other, and into close sympathy and more harmonious relations. They would thereby be better prepared to adopt uniform plans and modes. A course of instructions could be arranged and adopted that would probably be much better than that now in general use.

Uniformity in requirements for admission and graduation should be adopted as nearly as may be. Everything possible should be done to discourage a degrading competition for students. The competition should be in the thoroughness of the work done, in the way of instruction.

Let all the teachers who may be present meet at some convenient time during the meeting of the association and at least consider the matter.

